# Effects of undergraduates’ chronotypes and perceived stress on their sleep quality: A cross-sectional study

**DOI:** 10.1192/j.eurpsy.2023.1508

**Published:** 2023-07-19

**Authors:** E. C.-L. Lin, R.-R. Chen, J.-Y. Syu, J. C.-T. C. C.-T. Chen

**Affiliations:** 1 Department of Nursing, National Cheng Kung University (NCKU) and Hospital, Tainan, Taiwan, ROC, Tainan; 2 Psychiatric acute ward, Chiayi Branch, Taichung Veterans General Hospital, Chiayi, Taiwan, Province of China

## Abstract

**Introduction:**

Undergraduate students encounter developmental challenges during their transition into adulthood. Previous studies have claimed that adults with later chronotypes usually manifest negative psychological effects: poor sleep quality, greater stress, depression, and cognitive dysfunction. However, knowledge about the relationship between chronotype, stress, and sleep quality among young adults is lacking.

**Objectives:**

The present study investigated the relationship between undergraduates’ chronotypes and perceived stress on sleep quality.

**Methods:**

An online survey with a descriptive, cross-sectional design was conducted with a convenience sample of undergraduate students at a university in southern Taiwan. Those who were 20-25 years old and enrolled as a student were included; but who had been suspended or had deferred graduation were excluded. Students’ chronotype, stress, and sleep quality were assessed with three self-reported instruments: Munich Chronotype Questionnaire (MCTQ), Perceived Stress Scale (PSS), and Pittsburgh Sleep Quality Index (PSQI).

**Results:**

Of 161 undergraduates who completed the questionnaires, 51 reported using an alarm clock to wake and were removed from data analysis. One hundred and ten students’ mean age is 20.3 and perceived moderate stress. Sixty-one percent were poor-quality sleepers. The mean chronotype score was 5.7, and 85.5% had an intermediate chronotype, while 13.6% had an evening chronotype. Chronotype and perceived stress were positively correlated with sleep quality (p < .001). Social jetlag was positively correlated with chronotype (p =.036). Undergraduate’s later chronotype and higher stress perception predicted 30% of poorer sleep quality (p < .001).

**Conclusions:**

Undergraduate students’ chronotype and perceived stress were positively correlated and acted as predictors of the sleep quality. The findings could help to develop health-promotion interventions for these emerging adults to adjust their daily routines; and reduce their social jetlag, stress levels, and sleep disturbance.

**Disclosure of Interest:**

None Declared

